# The Comparative Virulence of the Tubercle Bacillus from Human and Bovine Sources1Read before the British Congress on Tuberculosis, Section on Pathology and Bacteriology, London, July 23-26, 1901.

**Published:** 1902-02

**Authors:** Mazyck P. Ravenel

**Affiliations:** Lecturer on Bacteriology, Veterinary Department, University of Pennsylvania; Bacteriologist of the State Live-Stock Sanitary Board of Pennsylvania


// THE JOURNAL

OF

COMPARATIVE MEDICINE AND

VETERINARY ARCHIVES.

VOL. XXIII. *FEBRUARY, 1902.* No. 2.

### THE COMPARATIVE VIRULENCE OF THE TUBERCLE BACILLUS FROM HUMAN AND BOVINE SOURCES.[1]

By MAZYCK P. RAVENEL, M.D.,

LECTURER ON BACTERIOLOGY, VETERINARY DEPARTMENT, UNIVERSITY OF PENNSYLVANIA; BACTERIOLOGIST OF THE STATE LIVE-STOCK SANITARY BOARD OF PENNSYLVANIA.

(*From the Laboratory of the State Live-stock Sanitary Board of Pennsylvania.*)

THE relation existing between the various types of the tubercle bacillus found in man and in the lower animals has been the subject of much discussion for several years past, and studies of the morphology, biology, and virulence have been undertaken with the object of determining whether or not constant differences could be detected in the organism from these two sources sufficient to justify their classification as distinct species.

From a purely practical stand-point the question is narrowed to a study of the bacillus as found in man and in cattle, and in the determination of the relation they bear to each other. The importance of this study is of value not only to the physician, anxious mainly for the protection of his clients, but to the general public, who look to the medical profession for guidance in such matters, and perhaps even more so for those who are concerned in the framing of laws and direction of public measures looking to the suppression of tuberculosis in man and animals. Efforts in this direction often meet with strong opposition, and extreme statements are made on very scanty evidence, which render the task of the hygienist much more difficult, and may result in the failure of his purpose. The lack of positive and authoritative knowledge in regard to this matter has in recent years led to the adoption of

[1] Read before the British Congress on Tuberculosis, Section on Pathology and Bacteriology, London, July 23–26, 1901.

retrograde measures in several places. The problem is one that can be entirely cleared up only by a long series of observations and carefully conducted experiments, which are tedious, exacting, and require a considerable lapse of time before results can be had. The correct interpretation of these results is in many cases not an easy matter.

The identity of the tubercle bacillus as found in the mammalia went unquestioned for many years, and laws for the prevention of the transmission of the bacillus of cattle to man have been based mainly on this belief. In America, Dr. Theobald Smith was the first to take up the systematic study of the bacilli isolated from various of the lower animals and man, and to call attention to certain fairly constant differences observed in the organisms from the two sources, which he considers sufficient to justify classifying them as distinct varieties or races, though his experiments "show unmistakably the close relationship existing among the various cultures studied."[1]

Dr. R. R. Dinwiddie,[2] Pathologist and Bacteriologist of the Arkansas Agricultural Experiment Station (U. S. A.), has carried out quite extensive experiments in which he has compared the virulence of the human and bovine tubercle bacillus, and also human tuberculous sputum with bovine material, for a number of the domestic animals, his results showing a greater power for both the bovine bacillus and material than for the human.

The vast practical importance of the matter, no less than its great scientific interest, was early realized by Dr. Leonard Pearson, State Veterinarian of Pennsylvania, and under his direction we have devoted much of our attention at the laboratory of the State Live-stock Sanitary Board for more than two years past to the study of the tubercle bacillus obtained from various cases of tuberculosis in man and in cattle.

In the carrying out of this work I have had throughout the efficient assistance of Dr. S. H. Gilliland, on whom a large share of the labor has fallen, and to whom is due much of the credit in its successful issue. To him and to the several physicians who have interested themselves in obtaining material for us I beg to express my obligation. During a considerable portion of the time that Part II. of the experiment was in progress Dr. W. G. Shaw was in charge of the animals, and did much of the work; while the bovine material for the inoculations was obtained and prepared by Dr. J. J. Repp, both of whom were at the time connected with the laboratory.

Our experimental work has been divided into two parts:

1. Isolation and study of pure cultures from various sources in man and cattle.

2. Testing the pathogenic power of tuberculous material of human and bovine origin.

1. Under the first division thirteen cultures, seven of human and six of bovine origin, are included in this report. Of these two human and two bovine have been compared under conditions as nearly identical throughout as it was possible to obtain them.

Cultures H (bovine) and K (human) were isolated within a few days of each other, while L (bovine) and M (human) were made on the same day. The first sub-cultures of both pairs were made on the same day, and all transfers since have been made simultaneously. The comparative pathogenicity of these cultures is shown in Tables I. and II.

Cultures H and K were tested also for puppies and pigs by feeding, as described below.

Culture H was recovered from the horse, goat, two puppies, two hogs, and man; while culture K was recovered only from two puppies and two hogs, the absence of lesions in other animals inoculated making it impossible to obtain a series comparable to culture H. The virulence of these recovered cultures, with the exception of that from the horse, was tested for guinea-pigs and rabbits, the results being given in Table III.

Human cultures I, J, U, and W, and bovine cultures F, Q, QQ, and T, were isolated as occasion arose, and their virulence tested only for guinea-pigs and rabbits. The dose and mode of inoculation were uniform in all cases, and the animals were kept under the same conditions, but beyond this the tests are not entirely parallel. The results are given in Table IV.

In addition to those cultures here mentioned we have isolated and examined nine others, morphologically and culturally. These have been taken into account in forming our conclusions, as well as the very careful descriptions given by Dr. Theobald Smith of the cultures studied by him.

The terms "human" and "bovine" have been applied to cultures to denote their origin from man or from cattle. Each culture is designated by a letter of the alphabet, and the generation indicated by a small figure at the lower right corner. Cultures which have been inoculated and recovered retain their original letter, a second letter inclosed in brackets being added to mark the recovered culture.

Table I.—Showing Virulence of Bovine Culture H.

| Culture. | Age, days. | Animals. | Date of inoculation. | Method of inoculation. | Dose in c.c. | Initial weight. | Weight at death. | Loss or gain in weight. | Died. | Killed. | Length of life. | Average. |
|---|---|---|---|---|---|---|---|---|---|---|---|---|
| $H_2$ | 15 | Guinea-pig | Sept. 2 | Subcutaneous | 1 | 290 gm. | 174 gm. | 116 gm. loss | Sept. 25 | ........... | 23 days | |
| $H_2$ | 15 | " " | " 2 | " | 1 | 310 " | 224 " | 86 " " | Oct. 9 | ........... | 37 " | 28⅓ days |
| $H_2$ | 15 | " " | " 2 | " | 1 | 300 " | 200 " | 100 " " | Sept. 27 | ........... | 25 " | |
| $H_2$ | 15 | Rabbit | " 2 | " | 1 | 1730 " | ........... | ............... | Nov. 7 | ........... | 66 " | 56½ " |
| $H_2$ | 15 | " | " 2 | " | 1 | 1580 " | ........... | ............... | Oct. 19 | ........... | 47 " | |
| $H_2$ | 29 | Dog | " 16 | Intrapulmonary | 2 | 20 lbs. | 18 lbs. | 2 lbs. loss | Nov. 14 | ........... | 59 " | 62½ " |
| $H_2$ | 29 | " | " 16 | " | 2 | 60 " | ........... | ............... | Nov. 21 | ........... | 66 " | |
| $H_3$ | 73 | Horse | Dec. 9 | " | 14 | 1030 " | 990 lbs. | 40 lbs. loss | ........... | June 25, 1900 | 6 m. 16 d. | |
| $H_3$ | 73 | Goat | " 9 | " | 4 | 79 " | 61 " | 18 " " | Dec. 31 | ........... | 22 days | 24 " |
| $H_3$ | 73 | " | " 9 | " | 4 | 29 " | 20¼ " | 8¾ " " | Jan. 4, 1900 | ........... | 26 " | |

Showing Virulence of Human Culture K.

| Culture. | Age, days. | Animals. | Date of inoculation. | Method of inoculation. | Dose in c.c. | Initial weight. | Weight at death. | Loss or gain in weight. | Died. | Killed. | Length of life. | Average. |
|---|---|---|---|---|---|---|---|---|---|---|---|---|
| $K_2$ | 15 | Guinea-pig | Sept. 2 | Subcutaneous | 1 | 380 gm. | 270 gm. | 110 gm. loss | Oct. 14 | ........... | 42 days | |
| $K_2$ | 15 | " " | " 2 | " | 1 | 320 " | 175 " | 145 " " | Sept. 30 | ........... | 28 " | 38 days |
| $K_2$ | 15 | " " | " 2 | " | 1 | 310 " | 215 " | 95 " " | Oct. 16 | ........... | 44 " | |
| $K_2$ | 15 | Rabbit | " 2 | " | 1 | 1375 " | 1970 " | 595 " gain | ........... | May 25, 1900 | 8 m. 23 d. | 8 m. 23 d. |
| $K_2$ | 15 | " | " 2 | " | 1 | 1500 " | 1820 " | 320 " " | ........... | " 25, " | 8 m. 23 d. | |
| $K_2$ | 29 | Dog | " 16 | Intrapulmonary | 2 | 21 lbs. | 20 lbs. | 1 lb. loss | ........... | Apr. 20, " | 7 m. 4 d. | 6 m. 19 d. |
| $K_2$ | 29 | " | " 16 | " | 2 | 57 " | 54 " | 3 lbs. " | ........... | Mar. 19, " | 6 m. 3 d. | |
| $K_3$ | 79 | Horse | Dec. 9 | " | 14 | 1275 " | 1370 " | 95 " gain | ........... | June 25, " | 6 m. 17 d. | |
| $K_3$ | 79 | Goat | " 9 | " | 4 | 73 " | 75 " | 2 " " | ........... | " 29, " | 6 m. 21 d. | 6 m. 21 d. |
| $K_3$ | 79 | " | " 9 | " | 4 | 28 " | 37 " | 9 " " | ........... | " 29, " | 6 m. 21 d. | |

Table II.—Showing Virulence of Bovine Culture L.

| Culture. | Age, days. | Animals. | Date of inoculation. | Method of inoculation. | Dose in c.c. | Initial weight. | Weight at death. | Loss or gain in weight. | Died. | Killed. | Length of life. | Average. |
|---|---|---|---|---|---|---|---|---|---|---|---|---|
| $L_2$ | 35 | Guinea-pig | Dec. 22 | Subcutaneous | 1 | 330 gm. | 210 gm. | 120 gm. loss | Jan. 13, 1900 | ........... | 22 days | |
| $L_2$ | 35 | " " | " 22 | " | 1 | 424 " | 240 " | 184 " " | " 13, " | ........... | 22 " | 26⅔ days |
| $L_2$ | 35 | " " | " 22 | " | 1 | 286 " | 246 " | 40 " " | " 27, " | ........... | 36 " | |
| $L_2$ | 35 | Rabbit | " 22 | " | 1 | 1720 " | 1216 " | 504 " " | Mar. 27, " | ........... | 95 " | 96½ " |
| $L_2$ | 35 | " | " 22 | " | 1 | 2100 " | 1450 " | 650 " " | " 30, " | ........... | 98 " | |
| $L_2$ | 35 | Dog | " 22 | Intrapulmonary | 3 | 31 lbs. | 30½ lbs. | 1½ lbs. " | ........... | Apr. 30, 1900 | 129 " | 130 " |
| $L_2$ | 35 | " | " 22 | " | 3 | 25½ " | 27 " | 1½ " gain | ........... | May 2, " | 131 " | |
| $L_2$ | 36 | Goat | " 23 | " | 4½ | 83 " | 73 " | 10 " loss | Jan. 8, 1900 | ........... | 16 " | |

Showing Virulence of Human Culture M.

| Culture. | Age, days. | Animals. | Date of inoculation. | Method of inoculation. | Dose in c.c. | Initial weight. | Weight at death. | Loss or gain in weight. | Died. | Killed. | Length of life. | Average. |
|---|---|---|---|---|---|---|---|---|---|---|---|---|
| $M_2$ | 35 | Guinea-pig | Dec. 22 | Subcutaneous | 1 | 350 gm. | 260 gm. | 90 gm. loss | Feb. 14, 1900 | ........... | 54 days | |
| $M_2$ | 35 | " " | " 22 | " | 1 | 410 " | 300 " | 110 " " | Jan. 13, " | ........... | 22 " | 34 days |
| $M_2$ | 35 | " " | " 22 | " | 1 | 300 " | 216 " | 84 " " | " 17, " | ........... | 26 " | |
| $M_2$ | 35 | Rabbit | " 22 | " | 1 | 1620 " | 1740 " | 120 " gain | ........... | May 25, 1900 | 154 " | 154 " |
| $M_2$ | 35 | " | " 22 | " | 1 | 1730 " | 1930 " | 200 " " | ........... | " 25, " | 154 " | |
| $M_2$ | 35 | Dog | " 22 | Intrapulmonary | 3 | 29¼ lbs. | 22 lbs. | 7¼ lbs. loss | ........... | Apr. 27, " | 126 " | 92½ " |
| $M_2$ | 35 | " | " 22 | " | 3 | 21 " | 18½ " | 2½ " " | Feb. 19, 1900 | ........... | 59 " | |
| $M_2$ | 36 | Goat | " 23 | " | 4½ | 52 " | 48 " | 4 " " | ........... | June 29, 1900 | 6 m. 6 d. | |

TABLE III.—SHOWING VIRULENCE OF BOVINE CULTURE H AND HUMAN CULTURE K FOR RABBITS AND GUINEA-PIGS, AFTER RECOVERY FROM ORIGINAL ANIMAL.

| Culture, | Recovered from | Age in days. | Animals. | Date of inoculation. | Initial weight. | Weight at death. | Loss or gain in weight. | Died. | Killed. | Length of life. | Average. |
|---|---|---|---|---|---|---|---|---|---|---|---|
| H (A)$_3$ | Goat | 22 | Guinea-pig | June 19 | 360 gm. | 225 gm. | 135 gm. loss | July 26 | .......... | 37 days | 27⅓ days. |
| H (A)$_3$ | " | 22 | " " | " 19 | 430 " | .......... | .......... | " 13 | .......... | 24 " | |
| H (A)$_3$ | " | 22 | " " | " 19 | 410 " | .......... | .......... | " 10 | .......... | 21 " | |
| H (B)$_2$ | Puppy | 42 | " " | May 28 | 280 " | 220 gm. | 60 gm. loss | " 2 | .......... | 35 " | 28⅓ " |
| H (B)$_2$ | " | 42 | " " | " 28 | 360 " | 210 " | 150 " " | June 23 | .......... | 26 " | |
| H (B)$_2$ | " | 42 | " " | " 28 | 260 " | 184 " | 76 " " | " 21 | .......... | 24 " | |
| H (C)$_2$ | " | 41 | " " | " 28 | 310 " | 210 " | 100 " " | July 2 | .......... | 35 " | 40 " |
| H (C)$_2$ | " | 41 | " " | " 28 | 295 " | 210 " | 85 " " | June 29 | .......... | 32 " | |
| H (C)$_2$ | " | 41 | " " | " 28 | 308 " | 230 " | 78 " " | July 20 | .......... | 53 " | |
| H (D)$_2$ | Hog | 36 | " " | Aug. 4 | 562 " | 380 " | 182 " " | Sept. 8 | .......... | 35 " | 33 " |
| H (D)$_2$ | " | 36 | " " | " 4 | 530 " | 260 " | 270 " " | " 7 | .......... | 34 " | |
| H (D)$_2$ | " | 36 | " " | " 4 | 470 " | 270 " | 200 " " | " 3 | .......... | 30 " | |
| H (E)$_2$ | Man (S.H.G.) | 27 | " " | " 2 | 460 " | 260 " | 200 " " | Aug. 29 | .......... | 27 " | 26⅓ " |
| H (E)$_2$ | " (S.H.G.) | 27 | " " | " 2 | 640 " | 400 " | 240 " " | Sept. 3 | .......... | 32 " | |
| H (E)$_2$ | " (S.H.G.) | 27 | " " | " 2 | 536 " | 370 " | 166 " " | Aug. 22 | .......... | 20 " | |
| H (F)$_2$ | Hog | 38 | " " | Sept. 13 | 610 " | 370 " | 240 " " | Oct. 29 | .......... | 46 " | 40 " |
| H (F)$_2$ | " | 38 | " " | " 13 | 330 " | 240 " | 90 " " | " 22 | .......... | 39 " | |
| H (F)$_2$ | " | 38 | " " | " 13 | 250 " | 200 " | 50 " " | " 18 | .......... | 35 " | |
| H (A)$_3$ | Goat | 22 | Rabbit | June 19 | 2030 " | 1680 " | 350 " " | July 18 | .......... | 29 " | 53 " |
| H (A)$_3$ | " | 22 | " | " 19 | 1800 " | 1380 " | 420 " " | Sept. 4 | .......... | 77 " | |
| H (B)$_2$ | Puppy | 42 | " | May 28 | 1455 " | 1390 " | 65 " " | Aug. 28 | .......... | 92 " | 84½ " |
| H (B)$_2$ | " | 42 | " | " 28 | 1975 " | 1180 " | 795 " " | " 13 | .......... | 77 " | |
| H (C)$_2$ | " | 41 | " | " 28 | 1770 " | 1050 " | 720 " " | " 11 | .......... | 75 " | 78½ " |
| H (C)$_2$ | " | 41 | " | " 28 | 1818 " | 1270 " | 548 " " | " 18 | .......... | 82 " | |
| H (D)$_2$ | Hog | 36 | " | Aug. 4 | .......... | 910 " | .......... | Sept. 12 | .......... | 39 " | 65 " |
| H (D)$_2$ | " | 36 | " | " 4 | .......... | 805 " | .......... | Nov. 3 | .......... | 91 " | |
| H (E)$_2$ | Man (S.H.G.) | 27 | " | " 2 | 2510 gm. | 1626 " | 884 gm. loss | Oct. 29 | .......... | 87 " | 61½ " |
| H (E)$_2$ | " (S.H.G.) | 27 | " | " 2 | 1870 " | 1620 " | 250 " " | Sept. 7 | .......... | 36 " | |
| H (F)$_2$ | Hog | 38 | " | Sept. 13 | 1530 " | 1190 " | 340 " " | Nov. 12 | .......... | 60 " | 63 " |
| H (F)$_2$ | " | 38 | " | " 13 | 840 " | 940 " | 100 " gain | " 18 | .......... | 66 " | |
| K (B)$_2$ | Puppy | 39 | Guinea-pig | May 28 | 315 " | 280 " | 35 " loss | Aug. 16 | .......... | 80 " | 63 " |
| K (B)$_2$ | " | 39 | " " | " 28 | 310 " | 270 " | 40 " " | July 26 | .......... | 59 " | |
| K (B)$_2$ | " | 39 | " " | " 28 | 330 " | 240 " | 90 " " | " 17 | .......... | 50 " | |
| K (C)$_2$ | " | 42 | " " | " 29 | 380 " | 220 " | 160 " " | " 27 | .......... | 59 " | 48 " |
| K (C)$_2$ | " | 42 | " " | " 29 | 440 " | 280 " | 160 " " | " 18 | .......... | 50 " | |
| K (C)$_2$ | " | 42 | " " | " 29 | 390 " | 220 " | 170 " " | " 3 | .......... | 35 " | |
| K (D)$_2$ | Hog | 29 | " " | Aug. 7 | 530 " | .......... | .......... | Sept. 8 | .......... | 32 " | 29½ " |
| K (D)$_2$ | " | 29 | " " | " 7 | 390 " | 330 gm. | 60 gm. loss | " 3 | .......... | 27 " | |
| K (E)$_2$ | " | 38 | " " | Sept. 13 | 350 " | 220 " | 130 " " | Oct. 16 | .......... | 33 " | 32⅓ " |
| K (E)$_2$ | " | 38 | " " | " 13 | 320 " | 260 " | 60 " " | " 9 | .......... | 26 " | |
| K (E)$_2$ | " | 38 | " " | " 13 | 310 " | 220 " | 90 " " | " 21 | .......... | 38 " | |
| K (B)$_2$ | Puppy | 39 | Rabbit | May 28 | 1445 " | 1360 " | 85 " " | Aug. 28 | .......... | 92 " | 104 " |
| K (B)$_2$ | " | 39 | " | " 28 | 1685 " | 1610 " | 75 " " | Sept. 21 | .......... | 116 " | |
| K (C)$_2$ | " | 42 | " | " 29 | 1560 " | 2010 " | 450 " gain | .......... | Oct. 4 | 128 " | 128 " |
| K (C)$_2$ | " | 42 | " | " 29 | 1560 " | 2610 " | 1050 " " | .......... | " 4 | 128 " | |
| K (D)$_2$ | Hog | 21 | " | Aug. 7 | .......... | .......... | .......... | Oct. 1 | .......... | 55 " | 71½ " |
| K (D)$_2$ | " | 21 | " | " 7 | .......... | .......... | .......... | Nov. 3 | .......... | 88 " | |
| K (E)$_2$ | " | 38 | " | Sept. 13 | 1260 gm. | 1290 gm. | 30 gm. gain | " 3 | .......... | 51 " | 62½ " |
| K (E)$_2$ | " | 38 | " | " 13 | 1050 " | 855 " | 195 " loss | " 26 | .......... | 74 " | |

### Table IV.—Showing Virulence of the Tubercle Bacillus from Various Sources for Rabbits and Guinea-pigs.

| Culture. | Age in days. | Animal. | Initial weight. | Weight at death. | Loss or gain in weight. | Date of inoculation | Died. | Killed. | Length of life. | Average. |
|---|---|---|---|---|---|---|---|---|---|---|
| $J_6$ Human . . . . | 20 | Guinea-pig | 610 gm. | 370 gm. | 240 gm. loss | July 16 | Sept. 24 | ........... | 77 days | |
| $J_6$ " . . . . | 20 | " " | 514 " | 320 " | 194 " " | " 16 | Oct. 1 | ........... | 77 " | 79$\frac{2}{3}$ days. |
| $J_6$ " . . . . | 20 | " " | 434 " | 310 " | 124 " " | " 16 | " 16 | ........... | 92 " | |
| $J_6$ " . . . . | 20 | Rabbit | 920 " | 1450 " | 530 " gain | " 16 | ........... | Dec. 19 | 156 " | |
| $J_6$ " . . . . | 20 | " | 855 " | 1470 " | 615 " " | " 16 | ........... | " 19 | 156 " | 156 " |
| $I_6$ " . . . . | 32 | Guinea-pig | 695 " | 510 " | 185 " loss | " 16 | Aug. 4 | ........... | 19 " | |
| $I_6$ " . . . . | 32 | " " | 700 " | 420 " | 280 " " | " 16 | Sept. 12 | ........... | 58 " | 54$\frac{1}{3}$ " |
| $I_6$ " . . . . | 32 | " " | 454 " | 360 " | 94 " " | " 16 | Oct. 10 | ........... | 86 " | |
| $I_6$ " . . . . | 32 | Rabbit | 900 " | 1850 " | 950 " gain | " 16 | ........... | Dec. 19 | 156 " | |
| $U_3$ " . . . . | 24 | Guinea-pig | 390 " | 210 " | 180 " loss | Jan. 14 | Feb. 20 | ........... | 37 " | |
| $U_3$ " . . . . | 24 | " " | 360 " | 290 " | 70 " " | " 14 | " 21 | ........... | 38 " | 43 " |
| $U_3$ " . . . . | 24 | " " | 330 " | 265 " | 65 " " | " 14 | Mar. 9 | ........... | 54 " | |
| $U_3$ " . . . . | 24 | Rabbit | 1710 " | 2040 " | 330 " gain | " 14 | ........... | June 15 | 152 " | |
| $U_3$ " . . . . | 24 | " | 1490 " | 2130 " | 640 " " | " 14 | ........... | " 15 | 152 " | 152 " |
| $W_3$ " . . . . | 19 | Guinea-pig | 745 " | ........... | ..................... | ........... | ........... | ........... | ........... | |
| $W_3$ " . . . . | 19 | " " | 730 " | ........... | ..................... | ........... | ........... | ........... | ........... | All animals living at end of 60 days. |
| $W_3$ " . . . . | 19 | " " | 700 " | ........... | ..................... | ........... | ........... | ........... | ........... | |
| $W_3$ " . . . . | 19 | Rabbit | 1470 " | ........... | ..................... | ........... | ........... | ........... | ........... | |
| $W_3$ " . . . . | 19 | " | 1750 " | ........... | ..................... | ........... | ........... | ........... | ........... | |
| $F_6$ Bovine . . . . | 23 | Guinea-pig | 404 " | 320 gm. | 84 gm. loss | Jan. 19 | Sept. 7 | ........... | 50 days | |
| $F_6$ " . . . . | 23 | " " | 480 " | 320 " | 160 " " | " 19 | Aug. 16 | ........... | 28 " | 39$\frac{1}{3}$ days. |
| $F_6$ " . . . . | 23 | " " | 546 " | 340 " | 206 " " | " 19 | " 28 | ........... | 40 " | |
| $F_6$ " . . . . | 23 | Rabbit | 2350 " | 1380 " | 970 " " | " 19 | Nov. 23 | ........... | 127 " | |
| $F_6$ " . . . . | 23 | " | 910 " | 670 " | 240 " " | " 19 | " 22 | ........... | 126 " | 126$\frac{1}{2}$ " |
| $Q_4$ " . . . . | 35 | Guinea-pig | 380 " | 270 " | 110 " " | " 19 | Aug. 4 | ........... | 16 " | |
| $Q_4$ " . . . . | 35 | " " | 510 " | 420 " | 90 " " | " 19 | " 12 | ........... | 24 " | 24$\frac{2}{3}$ " |
| $Q_4$ " . . . . | 35 | " " | 800 " | 560 " | 240 " " | " 19 | " 22 | ........... | 34 " | |
| $Q_4$ " . . . . | 35 | Rabbit | 1580 " | 980 " | 600 " " | " 16 | Oct. 13 | ........... | 86 " | |
| $Q_4$ " . . . . | 35 | " | 895 " | 875 " | 20 " " | " 19 | Sept. 30 | ........... | 73 " | 79$\frac{1}{2}$ " |
| $QQ_5$ " . . . . | 25 | Guinea-pig | 560 " | 380 " | 180 " " | Aug. 4 | Aug. 22 | ........... | 18 " | |
| $QQ_5$ " . . . . | 25 | " " | 510 " | 370 " | 140 " " | " 4 | Sept. 3 | ........... | 30 " | 24 " |
| $QQ_5$ " . . . . | 25 | Rabbit | 1930 " | 1500 " | 430 " " | " 4 | Oct. 17 | ........... | 74 " | |
| $QQ_5$ " . . . . | 25 | " | 1340 " | 1080 " | 260 " " | " 4 | Aug. 11 | ........... | 7 " | |
| $T_3$ " . . . . | 35 | Guinea-pig | 390 " | 290 " | 100 " " | Sept. 11 | Oct. 21 | ........... | 40 " | |
| $T_3$ " . . . . | 35 | " " | 420 " | 265 " | 155 " " | " 11 | " 10 | ........... | 29 " | |
| $T_3$ " . . . . | 35 | " " | 455 " | 340 " | 115 " " | " 11 | " 21 | ........... | 40 " | 36$\frac{1}{3}$ " |
| $T_3$ " . . . . | 35 | Rabbit | 1210 " | 1090 " | 120 " " | " 11 | " 18 | ........... | 37 " | |
| $T_3$ " . . . . | 35 | " | 1090 " | 860 " | 230 " " | " 11 | Nov. 12 | ........... | 62 " | 49$\frac{1}{2}$ " |

TABLE V.—SHOWING RESULT OF INOCULATION OF ANIMALS WITH TUBERCULOUS MATERIAL FROM BOVINE AND HUMAN SOURCES.

| Material. | Reference No. | Animals. | Date of inoculation. | Method of inoculation. | Dose in c.c. | Initial weight. | Weight at death. | Loss or gain in weight. | Died. | Killed. | Length of life, days. | Average days. |
|---|---|---|---|---|---|---|---|---|---|---|---|---|
| Bovine . . | 9,838 | Horse | July 14 | Intrapulmonary | 10 | 1205 lbs. | 1010 lbs. | 195 lbs. loss | Sept. 6 | ............ | 54 | 60 |
| “ . . | 9,840 | “ [1] | July 30 | “ | 10 | 1075 “ | 1015 “ | 60 “ “ | ............ | Nov. 14, 1899 | 66 | |
| “ . . | 9,701 | Pig | July 14 | “ | 4 | 130 “ | 78 “ | 52 “ “ | Aug. 23 | ............ | 40 | 50¾ |
| “ . . | 9,702 | “ | July 14 | “ | 4 | 131 “ | 103 “ | 28 “ “ | Oct. 19 | ............ | 97 | |
| “ . . | 9,708 | “ [1] | July 30 | “ | 5 | 94 “ | 86 “ | 8 “ “ | Sept. 12 | ............ | 44 | |
| “ . . | 9,706 | “ [1] | July 30 | “ | 5 | 85 “ | 72 “ | 13 “ “ | Aug. 21 | ............ | 22 | |
| “ . . | 9,697 | Sheep | July 14 | “ | 5 | 66 “ | 49 “ | 17 “ “ | “ 27 | ............ | 41 | 38 |
| “ . . | 9,906 | “ [1] | July 30 | “ | 5 | 71 “ | 49 “ | 22 “ “ | “ 31 | ............ | 32 | |
| “ . . | 9,841 | Dog | July 15 | “ | 5 | 34½ “ | 24 “ | 10½ “ “ | Jan. 15, 1899 | ............ | 184 | 214½ |
| “ . . | 8,010 | “ [1] | July 30 | “ | 5 | 28 “ | ......... | ............ | ............ | Mar. 20, 1899 | 245 | |
| “ . . | 8,089 | Cat | July 18 | “ | 2.5 | 2508 gm. | 1695 gm. | 813 gm. loss | Aug. 18 | ............ | 31 | 25½ |
| “ . . | 8,001 | “ [1] | July 30 | “ | 5 | 1150 “ | 1043 “ | 107 “ “ | “ 19 | ............ | 20 | |
| Human . . | 8,008 | Horse | Aug. 27 | “ | 10 | 1205 lbs. | 1225 lbs. | 20 lbs. gain | ............ | Mar. 14, 1899 | 199 | 183½ |
| “ . . | 8,038 | “ | Sept. 27 | “ | 10 | 1200 “ | 1370 “ | 170 “ “ | ............ | Mar. 14, “ | 168 | |
| “ . . | 9,704 | Pig | Aug. 27 | “ | 5 | 80 “ | 100 “ | 20 “ “ | Dec. 9 | ............ | 104 | 58⅓ |
| “ . . | 9,842 | “ | Aug. 27 | “ | 5 | 91 “ | 91 “ | ............ | Nov. 8 | ............ | 73 | |
| “ . . | 9,844 | “ [2] | Nov. 9 | “ | 5 | 70 “ | 71 “ | 1 lb. gain | Dec. 14 | ............ | 35 | |
| “ . . | 8,095 | “ [3] | Dec. 14 | “ | 5 | ......... | 193 “ | ............ | ............ | Mar. 21, 1899 | 97 | |
| “ . . | 9,705 | “ [2] | Nov. 9 | “ | 5 | 87 lbs. | 107 “ | 20 lbs. gain | Dec. 7 | ............ | 28 | |
| “ . . | 8,094 | “ [4] | Dec. 7 | “ | 5 | ......... | ......... | ............ | “ 20 | ............ | 13 | |
| “ . . | 9,698 | Sheep | Aug. 27 | “ | 5 | 70 lbs. | 74 lbs. | 4 lbs. gain | ............ | Mar. 17, 1899 | 202 | 202 |
| “ . . | 9,699 | “ | Aug. 27 | “ | 5 | 58 “ | 79 “ | 21 “ “ | ............ | Mar. 17, “ | 202 | |
| “ . . | 12,179 | Dog | Sept. 8 | “ | 5 | 55 “ | 28 “ | 27 “ loss | ............ | Mar. 25, “ | 229 | 146 |
| “ . . | 12,180 | “ | Aug. 27 | “ | 5 | 29 “ | 32 “ | 3 “ gain | Oct. 29 | ............ | 63 | |
| “ . . | 10,023 | Cat | Aug. 27 | “ | 2.5 | 2190 gm. | ......... | ............ | Aug. 29 | ............ | | |
| “ . . | 10,060 | “ | Sept. 8 | “ | 5 | ......... | ......... | ............ | Mar. 20, 1899 | ............ | 193 | 132½ |
| Human sputum | 5,984 | Calf | July 29 | Intraperitoneally | 10 | 108 gm. | 258 lbs. | 150 lbs. gain | ............ | Jan. 14, 1899 | 169 | |
| “ “ . | 8,499 | “ | July 29 | “ | 10 | 131 “ | 265 “ | 134 “ “ | ............ | Jan. 14, “ | 169 | |
| “ “ . | 8,049 | “ | May 16 | “ | 10 | ......... | 340 “ | ............ | ............ | Aug. 1, 1898 | 77 | |
| “ “ . | 8,050 | “ | May 16 | “ | 10 | ......... | 190 “ | ............ | ............ | Aug. 1, “ | 77 | |
| “ “ . | 8,074 | “ | Oct. 18 | Fed eleven times | 30-60 | 224 gm. | 385 “ | 161 lbs. gain | ............ | Feb. 18, 1899 | 123 | |
| “ “ . | 8,096 | “ | Oct. 18 | “ “ “ | 30-60 | 230 “ | 350 “ | 120 “ “ | ............ | Feb. 18, “ | 123 | |
| “ . . | 9,843 | “ [5] | Aug. 2 | Intraperitoneally | 20 | 108 “ | ......... | ............ | ............ | Jan. 10, “ | 161 | |
| “ . . | 9,846 | “ [6] | Aug. 2 | “ | 20 | 132 “ | ......... | ............ | ............ | Jan. 10, “ | 161 | |

[1] These animals were inoculated with the tissues of guinea-pigs which had died of general tuberculosis due to inoculation with material from a tuberculous cow. This material was the same as that used for the inoculation of the other animals. [2] Inoculated from tissues of pig 9842. [3] Inoculated from tissues of pig 9844. [4] Inoculated from tissues of pig 9705. [5] Inoculated from tissues of calf 8049. [6] Inoculated from tissues of calf 8050.

2. The second part of the work has been the inoculation of animals with tuberculous material from man and cattle. The plan and methods are described in another section, while the results are given in Tables V. and VI. As a part of this experiment human tuberculous sputum has been fed to two calves, and four have been inoculated with the same material. These have been included in Table V. and described further on.

Table VI.—Showing Comparative Extent of Lesions Produced by Inoculation of Human and Bovine Material.

| | Bovine material direct. | Bovine material passed through guinea-pigs. | Human material direct. | Human material passed through other animals. |
|---|---|---|---|---|
| Horses | Extensive disease of thoracic cavity. | Extensive involvement of lungs. | One horse limited involvement of one lung; the other was normal throughout. | Rapidly fatal, very acute process confined mainly to lungs; slower process in one, confined to lung & pleura of one side. |
| | Extensive disease of thoracic cavity. | Extensive disease of thoracic cavity. | Extensive disease of thoracic cavity. | |
| Sheep | Extensive disease of thoracic cavity. | Extensive disease of thoracic cavity. | Limited involvement of one lung and pleura of same side in one animal; the other normal throughout. | |
| Dogs | Extensive disease of thoracic cavity. | Limited involvement of one lung. | Limited involvement of pleura and pericardium in one animal; in the other extensive disease of the thoracic cavity. | |
| Cats | Extensive disease of thoracic cavity and spleen. | Extensive disease of thoracic cavity and mesenteric glands. | Normal throughout. | |

### Part I.—Isolation and Study of Pure Cultures from Various Sources in Man and Cattle.

*Method of Isolating Pure Cultures.* The isolation of cultures has been obtained by the method devised by Dr. Theobald Smith,[3] the principal features of which are: (1) The test-tubes, which are fitted with a cap like that of the well-known Miquel flask, ground on. The small tubulation in the cap is plugged with glass-wool. (2) The medium, which is dog's serum. The animal is bled with the strictest aseptic precautions, and the blood conducted to sterile jars through sterile rubber tubes. The serum is drawn off with pipettes and distributed at once into the test-tubes, put in the hardening oven, and coagulated at a low temperature (76° C.). Prepared in this way the serum needs no sterilization, and is much softer than when a higher temperature is employed. (3) The tubes are kept always inclined. (4) The tuberculous tissue selected for planting in our culture tubes is not crushed or rubbed over the surface, as advised in other methods. Young nodules from the omentum, spleen, liver, or a lymphatic gland are cut out, and a block of considerable size is placed on the surface of the serum. After remaining from two to three weeks in the incubator at 37.5° to 39° C. the tissue is pressed against the sides of the tubes with a stout platinum wire, and rubbed over the surface of the medium. Examination of the fluid squeezed out of the tissue at this time will generally indicate the final result. The tubes are replaced in the incubator, and within a week or ten days colonies of the tubercle bacillus usually appear. The nodules for inoculation of the tubes are best obtained from guinea-pigs, which are to be inoculated with the original matter from which we wish to obtain cultures. As soon as these show marked illness they are chloroformed and the cultures made. In general terms, the more recent the lesions the greater the chances of successful culture. As a rule, guinea-pigs can be killed in from three to four weeks after inoculation. If our material is of human origin the inoculation should be intraperitoneal; if bovine, subcutaneous inoculation will generally cause a sufficiently rapid involvement of the organs. (5) The incubator should contain a dish of water, to insure an abundance of moisture in the atmosphere. With the same object in view, it should be opened as seldom as possible.

The only modification of the above method in our work has been the addition of a 50 per cent. solution of glycerin in water to the serum in such proportion that the resulting mixture contains 5 per cent. of glycerin. This seems to make coagulation slightly more tardy; but the serum does not dry out as quickly, and the growth of the tubercle bacillus is facilitated. Serum prepared in this manner has been employed throughout for the cultures used in our inoculations.

*Preparation of Cultures for Inoculation, and Dosage.* The course of an experimental tuberculosis depends so largely on the number of bacilli introduced that exact dosage is an important factor in comparative work. Weighing naturally suggested itself, but a trial soon convinced us of its impracticability. The growth of human cultures is usually so abundant by the second generation that it is easy to obtain fairly dry masses which can be weighed quite accurately; but bovine cultures often grow very scantily for many generations as a thin film resembling ground glass. We have adopted as a uniform practice the method of Dr. Theobald

Smith—the use of a suspension of a given opacity. A portion of the growth is removed to the wall of a dry and sterile test-tube, and rubbed round and round with a glass rod until no lumps can be seen. Bouillon is then added, and after stirring thoroughly the mixture is allowed to stand quiet for two or three hours, so that the clumps may settle. The upper portion is poured off, and bouillon added until the suspension equals in turbidity a twenty-four-hour-old culture of the typhoid bacillus. The test culture is killed with formalin vapor and sealed, so that it can be used as a standard for a considerable time. Cover-glass preparations were made always as controls. In the tables below the generation of the cultures is indicated by the exponent, and the age of each culture is given in days in a separate column. For guinea-pigs and rabbits the dose has been throughout 1 c.c. of the suspension, introduced under the skin of the abdomen. For the other animals the dose and mode of inoculation are given in the tables.

*Morphology and Cultural Characteristics of Bacilli Examined.* The morphology of the bacilli in cultures of bovine origin is more uniform and constant than in cultures from man. The bovine bacilli are short, seldom more than 2 $\mu$ in length, and averaging less. In early generations many are seen which are oval, their length not more than double their breadth. They are thick and straight. They stain with carbol-fuchsin evenly and deeply, and beading is markedly absent, even in old cultures.

The human bacilli are, as a rule, much longer from the start, and tend to increase in length rapidly in sub-cultures. They are generally more or less curved, and some cultures contain many S-shaped forms, as culture M, noted below. They stain with carbol-fuchsin less deeply, and beading is a marked characteristic, often seen in the earliest growth.

These characteristics are most persistent in cultures on blood-serum. On glycerin-agar, glycerin-bouillon, and potato with glycerin the bacilli from the two sources approach each other in appearance and morphology much more closely.

The human cultures isolated have without exception grown more luxuriantly than the bovine cultures, though in at least two instances (cultures U and BB) the first growth was very long in appearing. This was due no doubt to their feeble virulence and consequent slow production of lesions in the tissues of guinea-pigs. A saprophytic life once established, they grew vigorously.

The bovine cultures are apt to grow as discrete colonies in the first culture, and for several generations are likely to grow as an exceedingly thin layer over a part of the medium, resembling closely ground glass.

The human bacillus can usually be induced to grow on glycerin-agar in sub-cultures made from the original growth on blood-serum. All attempts to obtain a like result with the bovine organism have failed.

Three of the bovine cultures examined have been isolated from milk. Two of these, L and Q, have shown throughout the characters of the type described as "bovine," while T approaches the human bacillus in morphology, being long and slender. It is, however, more virulent than any human culture tested.

Among the human cultures only one (M) has been isolated from sputum. Through the kindness of Dr. Alfred Hand, pathologist of the Children's Hospital, to whom we beg to make grateful acknowledgments, we have been able to obtain cultures from three cases of tuberculosis in young children in whom the intestine was involved. One of these, from which culture BB was isolated, was considered by Dr. Hand to be, "more clearly than any other he had ever seen, of intestinal origin," and was therefore studied with peculiar interest. Its pathogenicity has not yet been determined, but the course of the disease in the guinea-pigs inoculated to obtain the culture indicated a very feeble virulence, one living sixty-six and another ninety-six days. The bacilli, even in the first generation, are unusually long and thick. They stain deeply with carbol-fuchsin, but are beaded in a striking manner, the brightly stained portions being quite regularly disposed along the rod. They are most unlike what we have described as the bovine type in every way.

Cultures U and W, also from children with intestinal lesions, correspond to the human type in every particular, both as regards morphology and virulence.

### Comparison of Cultures H (Bovine) and K (Human). (Table I.)

*History of Cultures.* Culture H (bovine) was isolated from the mesenteric gland of a Jersey grade cow, about seven years old, which had been slaughtered for beef, but found to be extensively diseased and condemned. The lungs were the seat of an extensive and long-continued tuberculosis. Most of the foci had undergone caseation. The liver was involved also, but to a less extent. The gland from which the culture was obtained had undergone cheesy degeneration. On May 17, 1899, guinea-pigs were inoculated intraperitoneally with an emulsion made from the centre of this gland. On July 6th one pig was killed and cultures made on dog's serum. On July 28th the first growth was observed, and on August 18th sub-cultures were made from the omentum. Growth was fairly abundant. The bacilli were short, thick, straight, and stained evenly and deeply.

Culture K (human) was isolated from the lung of an adult negro man. The disease was not severe and was confined almost entirely to the lungs. Death was due to heart trouble. Guinea-pigs were inoculated intraperitoneally with an emulsion made from nodules taken from the lungs on June 10, 1899. On June 28th a pig was killed and cultures made on dog's serum. Growth was first observed on July 29th, the cultures from the spleen being used for sub-cultures, the first of which was made on August 18th. This culture grew rapidly and abundantly from the beginning. The bacilli were quite long, slender, and curved. They stained somewhat faintly, and showed a marked tendency to beading.

### Comparison of Cultures L (Bovine) and M (Human). (Table II.)

*History of Cultures.* Culture L (bovine) was obtained from the milk of a cow with tuberculosis of the udder. On May 22, 1899, a guinea-pig was inoculated with 10 c.c. of this milk. It died July 8th, and from it a second guinea-pig was inoculated intraperitoneally with an emulsion made from the spleen and omentum. On August 25th this pig was killed and two others inoculated intraperitoneally with an emulsion from the spleen and omentum. One of these was killed on September 18th and cultures made on dog's serum. On October 20th growth was first observed. On November 17th sub-cultures were made. The growth for several generations was very scanty. The bacilli were extremely short, many oval in shape. They took the stain evenly and deeply.

Culture M (human) was obtained from the sputum of a young adult female. Throat symptoms were most marked in this case. The disease was rapid and violent, death occurring in about five weeks after the sample of sputum was obtained. It contained large numbers of tubercle bacilli. On July 12, 1899, a guinea-pig was inoculated subcutaneously with the sputum. It was killed August 25th, and two pigs inoculated intraperitoneally with an emulsion made from the spleen and omentum. On September 18th a pig was killed and cultures made on dog's serum. Growth was first observed on October 15th, and sub-cultures were made on October 17th. Growth was rapid and abundant from the first, and the first transfers made from the original culture to glycerin-agar grew luxuriantly. The bacilli in the original culture were rather short, irregular in shape, many S-shaped, and, as a rule, stained quite evenly and deeply. In sub-cultures the bacilli soon became much longer, straighter, and showed beading.

*Summary and Post-mortem Notes.* It will be seen from the above tables that for each culture ten animals were used. Of the ten inoculated with culture H (bovine) nine died, while only one had to be killed, this being the horse; while of those inoculated with culture K (human) only three died, while seven had to be killed. With each of cultures L (bovine) and M (human) eight animals were inoculated. Of those inoculated with culture L six died and two were killed; while for culture M four died and four were killed. Taking the totals of these four cultures, eighteen animals were used for the bovine cultures and eighteen for the human. Of those inoculated with bovine tubercle bacillus fifteen died and three were killed; while of those inoculated with the human cultures seven died and eleven were killed. By making a closer study of the tables further differences in virulence between the bovine and human cultures may be brought out. All the guinea-pigs inoculated with those four cultures died, the extent of the post-mortem lesions being practically identical in all of them. Those inoculated with the human culture K lived almost ten days longer than those inoculated with the bovine culture H; while those inoculated with human culture M lived seven and one-third days longer than those inoculated with bovine culture L. All the rabbits inoculated with both of the human cultures had to be killed and showed no post-mortem lesions whatever, while those inoculated with the bovine cultures died, with extensive lesions. These lesions varied somewhat in the different animals, necrotic areas at the point of inoculation and complete involvement of the lungs being common to them all. The kidneys were also generally involved. Only one animal had generalized tuberculosis, involving the lungs, liver, kidneys, spleen, and omentum.

*Dogs.* The dogs showed a varying degree of susceptibility to all the cultures. Both of those inoculated with bovine culture H died, one in fifty-nine days, the other in sixty-six days. The smaller of the two had shown signs of illness for some days before death. The point of inoculation was well marked in the lung by a mass of tubercles 2.5 cm. in diameter. Both lungs were infiltrated throughout with gray nodules from 2 mm. to 4 mm. in diameter. Both pleural cavities contained a considerable amount of purulent effusion. The right pleura was roughened, without adhesions. The outer surface of the pericardium was thickly sown with minute nodules about 2 mm. in diameter, its interior being smooth. The liver was infiltrated throughout and necrotic, giving a nutmeg appearance. The right kidney contained a number of tubercular nodules ; the left was congested, but otherwise normal. The spleen had escaped infection. Through an accident no post-mortem was obtained of the other dog, but from his symptoms for some days before death it is reasonably certain that the disease was general, affecting particularly, perhaps, the lungs. Both of the dogs inoculated with human culture K had to be killed, the lesions in both being insignificant. In one the only marked change which could be found corresponded to the point of inoculation in the lung, where a small excavation was found almost completely filled in with scar tissue, radiating from the centre in star shape. Scrapings from cut surfaces of this area revealed the presence of tubercle bacilli. All other organs were entirely normal. In the other dog the point of inoculation was marked by a large nodule in the pleural surface of the lung. The surfaces of both lungs showed a great number of minute nodules. Scrapings from cut surfaces revealed tubercle bacilli in small numbers. On the external surface of the pericardium was a nodule, about one inch in diameter, which contained calcareous foci, incapsulated in dense fibrous material. The mediastinal glands were enlarged and contained a number of caseous foci. In all other respects the animal seemed normal.

*Horses.* A marked difference was noted in the effect of the cultures on the horses. The horse inoculated with bovine culture H showed no ill effects from the injection for some months after the injection. In the latter part of March, nearly four months after the injection, some emaciation could be noted, and the respirations were quickened, being on an average of twenty-two a minute and somewhat labored. On the first of April the temperature averaged 102.2° F. The symptoms grew very slightly more marked, and on June 25th, six months and a half after inoculation, the animal was killed. It had lost forty pounds in weight, though its condition was still fair. The costal pleuræ were extensively covered with masses of nodules of from 2 mm. to 18 mm.

in diameter, averaging nearly 12 mm. in thickness. These growths were somewhat more extensive on the left side than on the right, and tended to be more isolated and somewhat larger in size. The lungs were a grayish-pink, the bloodvessels being plainly outlined in a delicate network over the surface. They were covered with nodules, varying from 1 mm. up to 6 mm. of an inch in size, and closely packed. Both lungs were hard, dense, and devoid of elasticity, having much the feel of liver. At the point of inoculation on the right lung a small depression was found, filled in with a star-shaped cicatrix. This cicatricial tissue was surrounded with a nodule about 2.5 cm. in diameter, containing cheesy areas and much fibrous tissue. In the posterior part of the left lung a tubercular abscess about 2.5 cm. in diameter was found, and in the bottom of the lung several similar in size and character were seen. The bronchial glands were enlarged to twice their normal size, but showed no caseation. The surface of the pericardium was studded with minute nodules. The thoracic surface of the diaphragm was covered over a large portion of its surface with nodules the size of a pea, some of them pedunculated. Among the nodules were many shreds of fibrin, some of them 2.5 cm. in length. Similar masses were seen also on the costal surface of the lung. The liver, spleen, and kidneys appeared normal.

The horse inoculated with human culture K showed no clinical evidence of injury. The first of April its respiration was twelve a minute; its temperature was 100.5° F. It was killed on June 25th, about six months and a half after inoculation. Its condition was good. It had gained ninety-five pounds in weight. There was considerable fat subcutaneously and about the abdominal organs. All of the abdominal organs were entirely normal, with the exception of the liver, on the surface of which many fringes of fibrin were found. In the thoracic cavity both pleuræ were smooth and shiny, except at the point of inoculation on the right side, where a mass of small nodules with fringes of fibrin were seen. At the point of inoculation in the right lung was a cheesy abscess 4 cm. in diameter, and incapsulated with dense fibrous tissue, the walls being 6 mm. thick. Otherwise, the lung was entirely normal. In the left lung were three small tuberculous nodules about 12mm. in diameter, situated near the surface, and which had undergone caseation. The disease was evidently non-progressive, and was probably retrogressive.

*Goats.* Both the goats inoculated with bovine culture H died, one in twenty-two days and the other in twenty-six days, the lesions being much the same in both. There was thickening of the pleuræ and adhesion at the point of inoculation; otherwise they were normal. The lungs were covered with myriads of minute tubercles about 1 mm. in diameter, which gave a gritty feel to them. In the substance of the lung no tubercles could be found. The cut surfaces presented a pneumonic appearance, most marked in the smaller of the two animals. Scrapings from cut surfaces in any portion of the lung revealed enormous numbers of tubercle bacilli. The mucous in the bronchial tubes throughout the lungs also contained large numbers of tubercle bacilli. In the larger animal the liver, spleen, kidneys, peritoneum, and diaphragm were normal. The animal lost eighteen pounds in weight in the twenty-two days of its life after inoculation. The smaller goat showed involvement of the abdominal organs also. The omentum was studded with pearly tubercles 2mm. in diameter, one of which had undergone caseation. The surface of the spleen was thickly covered with tubercles 1 mm. in diameter. In the substance of the spleen no nodules could be found, but scrapings from cut surfaces showed a large number of tubercle bacilli. The liver showed no macroscopical tubercles, but scrapings from cut surfaces were rich in bacilli, and in sections under the microscope tubercles were seen, with a few giant cells. Microscopical examination of the lungs, however, revealed no tubercles. The infection in this animal was diffuse, and it is considered as a case of tubercular septicæmia.

Both of the goats inoculated with human culture K gained in weight and were killed after six months and twenty-one days. Both were in good condition, having a considerable amount of fat in the subcutaneous tissues and around the abdominal organs. In the larger animal at the point of inoculation there was an area of adhesion to the pleura about 8 mm. in diameter. On the surface of the left lung were two or three nodules about 3 mm. in diameter which had undergone caseation. This lung is attached at its lower edge to the diaphragm by fringes of fibrin, and along this edge a few nodules about 12 mm. in diameter and containing cheesy pus were found. The entire surface of the left lung was covered with fringes of fibrin of a reddish-pink color. In both lungs the elasticity was preserved, and neither revealed nodules either by palpation or on section. The left pleura was covered with a network of fibrin similar to that seen in the lung; the right was normal. The mediastinal glands were much enlarged and calcareous. In the liver two small nodules were found near the surface, the organ being otherwise normal. In the smaller animal the omentum contained upward of a hundred nodules from 2 to 12 mm. in diameter. The left lung was attached to the pleura at its edge by numerous bands of fibrin and at its posterior surface to the diaphragm in the same manner. Along the lower border were a number of nodules about 6 mm. in diameter which had undergone caseation. The right lung was normal. The mediastinal glands were enlarged and calcareous. The left pleura shows ten or twelve nodules 6 mm. in diameter, which are cheesy ; besides a large one, corresponding to the point of inoculation. The diaphragm is attached to the spleen and to the stomach by bands of fibrin, but no nodules were found. The liver was normal ; the portal glands were enlarged and contained many cheesy nodules. The left kidney contained a number of cheesy nodules. The right kidney was normal. The mesenteric glands were enlarged and several of them contained calcareous areas.

(To be continued.)

